# Sesquicaesium hemisodium tetra­cyanidoplatinate(II) sesquihydrate

**DOI:** 10.1107/S1600536810032551

**Published:** 2010-08-18

**Authors:** Andrew P. Weber, Milorad Stojanovic, Matthew R. Pischek, Richard E. Sykora

**Affiliations:** a Department of Chemistry, University of South Alabama, Mobile, AL 36688-0002, USA

## Abstract

The title compound, Cs_1.5_Na_0.5_[Pt(CN)_4_]·1.5H_2_O, was isolated from solution as a salt. The tetra­cyanidoplatinate (TCP) anions are stacked in a linear quasi-one-dimensional arrangement along the *b* axis, with Pt⋯Pt inter­actions of 3.6321 (5) Å. The mixed alkali metal TCP contains three distinct alkali metal positions in the structure that do not show any mixed occupancy: Cs1 (site symmetry 2), Cs2 (general position) and Na1 (site symmetry 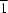). The Na^+^ ion contains an octa­hedral coordination environment composed of two water mol­ecules and four N-terminal cyanides, which serve to bridge TCP anions. The Cs^+^ cations contain mono- and bicapped square-prismatic environments, where the square prisms are formed from cyanide N atoms with water mol­ecules capping the faces. The 1.5 water mol­ecules per formula unit are a result of two fully occupied sites, one on a general position and one on a twofold rotation axis. Weak hydrogen-bonding inter­actions are observed between one water mol­ecule and terminal N-atom acceptors from TCP, while the second water mol­ecule is not involved in hydrogen bonding.

## Related literature

Crystalline TCP systems have been studied extensively for their inter­esting structural and spectroscopic, especially photoluminescent, properties (Holzapfel *et al.*, 1981 ▶; Gliemann & Yersin, 1985 ▶; Stojanovic *et al.*, 2010 ▶). An intrinsic factor affecting the optical properties and arrangement of TCP chains is the identity of the cations involved, and much work has been done in the systematic study of various combinations of alkali metal cations involved (Holzapfel *et al.*, 1981 ▶). However, only one known reference of a caesium/sodium mixed alkali metal TCP, *viz.* NaCs[Pt(CN)_4_]·1.5H_2_O, exists (Bergsoe *et al.*, 1962 ▶), which notes the synthesis and scintillation properties for the compound, but not any structural information.

## Experimental

### 

#### Crystal data

Cs_1.5_Na_0.5_[Pt(CN)_4_]·1.5H_2_O
*M* *_r_* = 1074.11
Monoclinic, 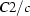

*a* = 17.4090 (5) Å
*b* = 7.2190 (1) Å
*c* = 18.3921 (5) Å
β = 117.858 (4)°
*V* = 2043.56 (11) Å^3^
*Z* = 4
Mo *K*α radiation
μ = 18.99 mm^−1^
*T* = 290 K
0.20 × 0.14 × 0.08 mm

#### Data collection

Oxford Diffraction Excalibur-E diffractometer
Absorption correction: multi-scan (*CrysAlis PRO*; Oxford Diffraction, 2010 ▶) *T*_min_ = 0.471, *T*_max_ = 1.00
8164 measured reflections
1949 independent reflections
1789 reflections with *I* > 2σ(*I*)
*R*_int_ = 0.019

#### Refinement

*R*[*F* ^2^ > 2σ(*F* ^2^)] = 0.014
*wR*(*F* ^2^) = 0.034
*S* = 1.15
1949 reflections
126 parameters
6 restraints
H atoms treated by a mixture of independent and constrained refinement
Δρ_max_ = 0.63 e Å^−3^
Δρ_min_ = −0.47 e Å^−3^

### 

Data collection: *CrysAlis PRO* (Oxford Diffraction, 2010 ▶); cell refinement: *CrysAlis PRO*; data reduction: *CrysAlis PRO*; program(s) used to solve structure: *SHELXS97* (Sheldrick, 2008 ▶); program(s) used to refine structure: *SHELXL97* (Sheldrick, 2008 ▶); molecular graphics: *OLEX2* (Dolomanov *et al.*, 2009 ▶); software used to prepare material for publication: *publCIF* (Westrip, 2010 ▶).

## Supplementary Material

Crystal structure: contains datablocks I, global. DOI: 10.1107/S1600536810032551/wm2385sup1.cif

Structure factors: contains datablocks I. DOI: 10.1107/S1600536810032551/wm2385Isup2.hkl

Additional supplementary materials:  crystallographic information; 3D view; checkCIF report

## Figures and Tables

**Table 1:** Hydrogen-bond geometry (Å, °)

*D*—H⋯*A*	*D*—H	H⋯*A*	*D*⋯*A*	*D*—H⋯*A*
O1—H1*A*⋯N1^i^	0.85 (2)	2.37 (3)	3.154 (6)	155 (5)
O1—H1*B*⋯N3^ii^	0.83 (2)	2.17 (2)	2.982 (5)	166 (5)

Symmetry codes: (i) 
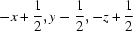
; (ii) 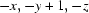.

**Fig. 1.**
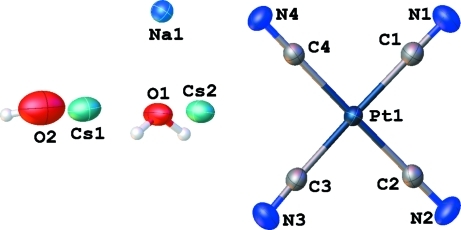
The asymmetric unit of Cs1.5Na0.5Pt(CN)4.1.5H2O with the atom-numbering scheme. Displacement ellipsoids for non-hydrogen atoms are drawn at the 50% probability level.

**Fig. 2.**
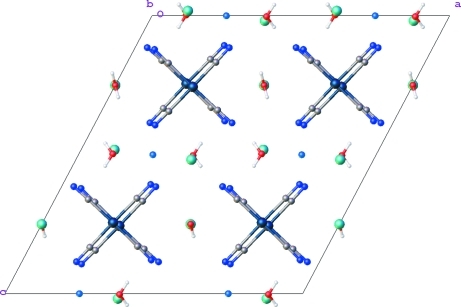
A packing diagram for Cs1.5Na0.5Pt(CN)4.1.5H2O along the b axis illustrating the tetracyanidoplatinate stacking.

## supplementary crystallographic information

### Comment

Crystalline tetracyanidoplatinate (TCP) systems have been studied extensively for their interesting structural and spectroscopic, especially photoluminescent, properties (Holzapfel *et al.*, 1981; Gliemann & Yersin, 1985; Stojanovic *et al.*, 2010). The title compound, Cs_1.5_Na_0.5_Pt(CN)_4_.1.5H_2_O, was obtained as an unexpected product from a reaction that was an attempt to prepare a heterometallic cyanidometalate complex containing Eu(III), TCP, and dicyanidoaurate moieties. A number of related mixed-metal cation (alkali, alkaline-earth) TCPs have been reported (Holzapfel *et al.*, 1981; Gliemann & Yersin, 1985). However, only one known reference of a caesium/ sodium mixed-alkali metal TCP (NaCsPt(CN)_4_.1.5H_2_O) exists (Bergsoe *et al.*, 1962), which notes the synthesis and scintillation properties for the compound, but not any structural information.

The structure of the title compound consists of pseudo one-dimensional chains of square-planar TCP anions tethered by Pt···Pt interactions of 3.6321 (5) Å. The platinate chains run parallel to the *b* axis and are bridged by ionic interactions among Cs^+^ and Na^+^ ions with the N atoms of the cyanidoplatinate, and show a loose packing of water molecules within that space. The water molecules feature weak H-bonding interactions with the N atoms of the platinate as well (Table 1). The Na^+^ ion contains a nearly regular octahedral coordination environment composed of two *trans* water molecules and four cyanido N atoms. Cs1 and Cs2 contain mono- and bi-capped square prismatic environments, respectively, where the slightly distorted square prisms are formed from cyanido N atoms and the capping positions are occupied by water molecules. As per each discrete TCP anion, the empirical structure of the compound contains an equivalency of 1.5 Cs^+^ as a result of the Cs1 site residing on a twofold rotation axis and Cs2 occupying a general position, 1.5 H_2_O molecules due to O1 residing on a general position and the presence of O2 on a twofold rotation axis, and 0.5 Na^+^ as a result of Na1 residing on an inversion center.

The N1 and N3 sites, *trans* to one another on the TCP anion, are involved in H-bonding interactions to the water molecule containing O1, while the other *trans* pair of cyanide groups containing N2 and N4 are involved in interactions with Na^+^ (2.527 (4) and 2.541 (4) Å, respectively). The O2 water molecule interacts with Cs1 at a distance of 3.103 (6) Å, but does not engage in any meaningful H-bonding interactions.

### Experimental

Eu(CF_3_SO_3_)_3_.9H_2_O (98%), Na_2_Pt(CN)_4_ (99.95%), NaAu(CN)_2_ (99.9%), and CsCl (99.9%) were used as received from Alfa Aesar. The title compound was obtained inadvertently in an attempt to produce a heterometallic cyanidometallate compound containing europium, TCP, and dicyanidoaurate. This involved the following preparation: Eu-trifluoromethanesulfonate (6.0 mg, 0.076 mmol) dissolved in 400 µl 80% CH_3_CN:H_2_O within a 5 ml test tube was layered with the solutions of sodium tetracyanidoplatinate (6.9 mg, 0.020 mmol) dissolved in 300 µl 80% CH_3_CN:H_2_O, and sodium dicyanidoaurate (2.7 mg, 0.010 mmol) dissolved in 400 µl 80% CH_3_CN:H_2_O. The solution was allowed to stand for 25 minutes until 1 ml caesium chloride (33.7 mg, 0.20 mmol) in 80% CH_3_CN:H_2_O solution was layered onto the mixture. Colorless, transparent crystals of Cs_1.5_Na_0.5_Pt(CN)_4_.1.5H_2_O were harvested from the reaction tube following slow evaporation of solvent.

### Refinement

All H-atoms on the water molecules were located in a difference map and restrained with O—H distances of 0.85 Å, H···H separations of 1.39 Å, and *U*_iso_(H) = 1.5*U*_eq_(O).

### Crystal data

**Table:**

Cs_1.5_Na_0.5_[Pt(CN)_4_]·1.5H_2_O	*F*(000) = 1864
*M**_r_* = 1074.11	*D*_x_ = 3.491 Mg m^−^^3^
Monoclinic, *C*2/*c*	Mo *K*α radiation, λ = 0.71073 Å
Hall symbol: -C 2yc	Cell parameters from 6424 reflections
*a* = 17.4090 (5) Å	θ = 3.1–25.6°
*b* = 7.2190 (1) Å	µ = 18.99 mm^−^^1^
*c* = 18.3921 (5) Å	*T* = 290 K
β = 117.858 (4)°	Prism, colorless
*V* = 2043.56 (11) Å^3^	0.20 × 0.14 × 0.08 mm
*Z* = 4	

### Data collection

**Table:**

Oxford Diffraction Excalibur-E diffractometer	1949 independent reflections
Radiation source: fine-focus sealed tube	1789 reflections with *I* > 2σ(*I*)
graphite	*R*_int_ = 0.019
Detector resolution: 16.0514 pixels mm^-1^	θ_max_ = 25.7°, θ_min_ = 3.1°
ω scans	*h* = −21→21
Absorption correction: multi-scan (*CrysAlis PRO*; Oxford Diffraction, 2010)	*k* = −8→8
*T*_min_ = 0.471, *T*_max_ = 1.00	*l* = −22→22
8164 measured reflections	

### Refinement

**Table:**

Refinement on *F*^2^	Secondary atom site location: difference Fourier map
Least-squares matrix: full	Hydrogen site location: difference Fourier map
*R*[*F*^2^ > 2σ(*F*^2^)] = 0.014	H atoms treated by a mixture of independent and constrained refinement
*wR*(*F*^2^) = 0.034	*w* = 1/[σ^2^(*F*_o_^2^) + (0.0147*P*)^2^ + 4.0939*P*] where *P* = (*F*_o_^2^ + 2*F*_c_^2^)/3
*S* = 1.15	(Δ/σ)_max_ = 0.002
1949 reflections	Δρ_max_ = 0.63 e Å^−^^3^
126 parameters	Δρ_min_ = −0.47 e Å^−^^3^
6 restraints	Extinction correction: *SHELXL97* (Sheldrick, 2008), Fc^*^=kFc[1+0.001xFc^2^λ^3^/sin(2θ)]^-1/4^
Primary atom site location: structure-invariant direct methods	Extinction coefficient: 0.000819 (19)

### Special details

**Table:**

Geometry. All e.s.d.'s (except the e.s.d. in the dihedral angle between two l.s. planes) are estimated using the full covariance matrix. The cell e.s.d.'s are taken into account individually in the estimation of e.s.d.'s in distances, angles and torsion angles; correlations between e.s.d.'s in cell parameters are only used when they are defined by crystal symmetry. An approximate (isotropic) treatment of cell e.s.d.'s is used for estimating e.s.d.'s involving l.s. planes.
Refinement. Refinement of *F*^2^ against ALL reflections. The weighted *R*-factor *wR* and goodness of fit *S* are based on *F*^2^, conventional *R*-factors *R* are based on *F*, with *F* set to zero for negative *F*^2^. The threshold expression of *F*^2^ > 2σ(*F*^2^) is used only for calculating *R*-factors(gt) *etc*. and is not relevant to the choice of reflections for refinement. *R*-factors based on *F*^2^ are statistically about twice as large as those based on *F*, and *R*- factors based on ALL data will be even larger.

### Fractional atomic coordinates and isotropic or equivalent isotropic displacement parameters (Å^2^)

**Table:**

	*x*	*y*	*z*	*U*_iso_*/*U*_eq_	
Cs1	0.0000	1.18745 (7)	−0.2500	0.04903 (13)	
Cs2	0.111975 (18)	0.76781 (4)	−0.017780 (19)	0.03819 (9)	
Na1	0.2500	0.2500	0.0000	0.0307 (5)	
Pt1	0.236863 (10)	0.51660 (2)	0.244311 (9)	0.02326 (7)	
C1	0.3610 (3)	0.5200 (6)	0.3297 (3)	0.0321 (10)	
C2	0.2026 (3)	0.5153 (5)	0.3330 (2)	0.0285 (9)	
C3	0.1120 (3)	0.5245 (6)	0.1605 (3)	0.0298 (9)	
C4	0.2736 (3)	0.5128 (5)	0.1564 (2)	0.0267 (9)	
N1	0.4315 (3)	0.5232 (6)	0.3789 (3)	0.0477 (11)	
N2	0.1834 (3)	0.5155 (6)	0.3851 (3)	0.0446 (10)	
N3	0.0407 (3)	0.5366 (6)	0.1146 (3)	0.0465 (10)	
N4	0.2955 (3)	0.5061 (5)	0.1069 (2)	0.0376 (9)	
O1	0.1138 (2)	0.2401 (5)	−0.0028 (2)	0.0443 (9)	
H1A	0.109 (3)	0.213 (8)	0.040 (2)	0.066*	
H1B	0.070 (2)	0.289 (7)	−0.040 (2)	0.066*	
O2	0.0000	0.7576 (8)	−0.2500	0.087 (2)	
H2	−0.031 (5)	0.698 (5)	−0.2927 (19)	0.131*	

### Atomic displacement parameters (Å^2^)

**Table:**

	*U*^11^	*U*^22^	*U*^33^	*U*^12^	*U*^13^	*U*^23^
Cs1	0.0443 (2)	0.0501 (3)	0.0628 (3)	0.000	0.0335 (2)	0.000
Cs2	0.02998 (15)	0.04395 (17)	0.04217 (17)	0.00112 (12)	0.01814 (13)	0.00518 (13)
Na1	0.0286 (12)	0.0374 (13)	0.0269 (12)	−0.0005 (10)	0.0135 (10)	0.0005 (10)
Pt1	0.02326 (9)	0.02732 (10)	0.01992 (9)	−0.00030 (6)	0.01069 (7)	−0.00112 (6)
C1	0.033 (2)	0.035 (2)	0.029 (2)	−0.0009 (19)	0.016 (2)	−0.0013 (19)
C2	0.028 (2)	0.028 (2)	0.027 (2)	−0.0014 (17)	0.0107 (18)	−0.0025 (17)
C3	0.032 (2)	0.034 (2)	0.026 (2)	−0.0021 (18)	0.0149 (19)	−0.0017 (18)
C4	0.025 (2)	0.026 (2)	0.026 (2)	0.0001 (17)	0.0094 (17)	−0.0009 (17)
N1	0.031 (2)	0.059 (3)	0.043 (2)	−0.0010 (19)	0.0084 (19)	−0.003 (2)
N2	0.059 (3)	0.050 (2)	0.038 (2)	−0.004 (2)	0.033 (2)	−0.0024 (19)
N3	0.032 (2)	0.056 (3)	0.042 (2)	−0.0005 (19)	0.0088 (19)	−0.004 (2)
N4	0.043 (2)	0.044 (2)	0.034 (2)	−0.0008 (18)	0.0247 (18)	−0.0023 (17)
O1	0.0303 (16)	0.066 (2)	0.038 (2)	0.0061 (16)	0.0175 (15)	0.0116 (17)
O2	0.083 (5)	0.069 (4)	0.127 (7)	0.000	0.063 (5)	0.000

### Geometric parameters (Å, °)

**Table:**

Cs1—O2	3.103 (6)	Pt1—C2	1.982 (4)
Cs1—N1^i^	3.464 (4)	Pt1—C1	1.991 (4)
Cs2—N1^ii^	3.191 (4)	Pt1—C3	1.993 (4)
Cs2—N4^i^	3.227 (4)	Pt1—C4	1.996 (4)
Cs2—N3^iii^	3.262 (4)	C1—N1	1.135 (6)
Cs2—N2^iv^	3.316 (4)	C2—N2	1.153 (6)
Cs2—O1^v^	3.420 (4)	C3—N3	1.132 (6)
Cs2—N4	3.491 (4)	C4—N4	1.141 (5)
Na1—O1	2.349 (3)	O1—H1A	0.85 (4)
Na1—N2^vi^	2.527 (4)	O1—H1B	0.83 (4)
Na1—N4	2.541 (4)	O2—H2	0.84 (3)
			
O2—Cs1—N1^i^	63.96 (7)	C4—Cs2—N2^ix^	63.43 (9)
O2—Cs1—N1^ii^	63.96 (7)	N1^ii^—Cs2—C3	110.04 (10)
N1^i^—Cs1—N1^ii^	127.93 (15)	N4^i^—Cs2—C3	153.14 (10)
O2—Cs1—N3^vii^	124.57 (7)	N3^iii^—Cs2—C3	80.06 (10)
N1^i^—Cs1—N3^vii^	169.41 (10)	N2^iv^—Cs2—C3	109.76 (10)
N1^ii^—Cs1—N3^vii^	61.00 (10)	O1^v^—Cs2—C3	113.80 (9)
O2—Cs1—N3^viii^	124.57 (7)	N4—Cs2—C3	62.76 (9)
N1^i^—Cs1—N3^viii^	61.00 (10)	N1^ix^—Cs2—C3	61.13 (9)
N1^ii^—Cs1—N3^viii^	169.41 (10)	N3—Cs2—C3	17.67 (9)
N3^vii^—Cs1—N3^viii^	110.86 (14)	C4—Cs2—C3	45.64 (9)
O2—Cs1—N4^ii^	67.01 (6)	N2^ix^—Cs2—C3	92.48 (9)
N1^i^—Cs1—N4^ii^	82.40 (9)	N1^ii^—Cs2—C1^ix^	89.08 (11)
N1^ii^—Cs1—N4^ii^	77.84 (9)	N4^i^—Cs2—C1^ix^	110.29 (9)
N3^vii^—Cs1—N4^ii^	106.38 (9)	N3^iii^—Cs2—C1^ix^	123.44 (10)
N3^viii^—Cs1—N4^ii^	99.28 (9)	N2^iv^—Cs2—C1^ix^	151.87 (10)
O2—Cs1—N4^i^	67.01 (6)	O1^v^—Cs2—C1^ix^	56.47 (9)
N1^i^—Cs1—N4^i^	77.84 (9)	N4—Cs2—C1^ix^	88.55 (9)
N1^ii^—Cs1—N4^i^	82.40 (9)	N1^ix^—Cs2—C1^ix^	17.65 (9)
N3^vii^—Cs1—N4^i^	99.28 (9)	N3—Cs2—C1^ix^	62.37 (9)
N3^viii^—Cs1—N4^i^	106.38 (9)	C4—Cs2—C1^ix^	73.70 (9)
N4^ii^—Cs1—N4^i^	134.02 (12)	N2^ix^—Cs2—C1^ix^	59.27 (9)
O2—Cs1—N2^viii^	125.63 (6)	C3—Cs2—C1^ix^	57.73 (9)
N1^i^—Cs1—N2^viii^	104.50 (9)	O1—Na1—O1^x^	180.00 (17)
N1^ii^—Cs1—N2^viii^	105.13 (9)	O1—Na1—N2^vi^	93.58 (13)
N3^vii^—Cs1—N2^viii^	65.76 (9)	O1^x^—Na1—N2^vi^	86.42 (13)
N3^viii^—Cs1—N2^viii^	75.49 (9)	O1—Na1—N2^iv^	86.42 (13)
N4^ii^—Cs1—N2^viii^	167.20 (9)	O1^x^—Na1—N2^iv^	93.58 (13)
N4^i^—Cs1—N2^viii^	58.66 (9)	N2^vi^—Na1—N2^iv^	180.00 (16)
O2—Cs1—N2^vii^	125.63 (6)	O1—Na1—N4^x^	90.84 (13)
N1^i^—Cs1—N2^vii^	105.13 (9)	O1^x^—Na1—N4^x^	89.16 (13)
N1^ii^—Cs1—N2^vii^	104.50 (9)	N2^vi^—Na1—N4^x^	90.85 (13)
N3^vii^—Cs1—N2^vii^	75.49 (9)	N2^iv^—Na1—N4^x^	89.15 (13)
N3^viii^—Cs1—N2^vii^	65.76 (9)	O1—Na1—N4	89.16 (13)
N4^ii^—Cs1—N2^vii^	58.66 (9)	O1^x^—Na1—N4	90.84 (13)
N4^i^—Cs1—N2^vii^	167.20 (9)	N2^vi^—Na1—N4	89.15 (13)
N2^viii^—Cs1—N2^vii^	108.74 (13)	N2^iv^—Na1—N4	90.85 (13)
O2—Cs1—C1^ii^	66.00 (7)	N4^x^—Na1—N4	180.00 (18)
N1^i^—Cs1—C1^ii^	126.47 (9)	C2—Pt1—C1	89.08 (17)
N1^ii^—Cs1—C1^ii^	17.94 (9)	C2—Pt1—C3	89.86 (17)
N3^vii^—Cs1—C1^ii^	63.92 (10)	C1—Pt1—C3	177.41 (17)
N3^viii^—Cs1—C1^ii^	154.32 (10)	C2—Pt1—C4	178.54 (16)
N4^ii^—Cs1—C1^ii^	61.35 (9)	C1—Pt1—C4	89.91 (17)
N4^i^—Cs1—C1^ii^	99.31 (9)	C3—Pt1—C4	91.18 (16)
N2^viii^—Cs1—C1^ii^	119.49 (9)	N1—C1—Pt1	179.2 (4)
N2^vii^—Cs1—C1^ii^	88.95 (9)	N1—C1—Cs1^i^	70.0 (3)
O2—Cs1—C1^i^	66.00 (7)	Pt1—C1—Cs1^i^	110.74 (16)
N1^i^—Cs1—C1^i^	17.94 (9)	N1—C1—Cs2^vi^	69.5 (3)
N1^ii^—Cs1—C1^i^	126.47 (9)	Pt1—C1—Cs2^vi^	110.15 (16)
N3^vii^—Cs1—C1^i^	154.32 (10)	Cs1^i^—C1—Cs2^vi^	110.37 (11)
N3^viii^—Cs1—C1^i^	63.92 (10)	N2—C2—Pt1	179.4 (4)
N4^ii^—Cs1—C1^i^	99.31 (9)	N2—C2—Cs1^vii^	75.9 (3)
N4^i^—Cs1—C1^i^	61.35 (9)	Pt1—C2—Cs1^vii^	104.31 (14)
N2^viii^—Cs1—C1^i^	88.95 (9)	N2—C2—Cs2^vi^	73.6 (3)
N2^vii^—Cs1—C1^i^	119.49 (9)	Pt1—C2—Cs2^vi^	106.10 (15)
C1^ii^—Cs1—C1^i^	132.01 (13)	Cs1^vii^—C2—Cs2^vi^	148.78 (12)
O2—Cs1—C3^viii^	123.99 (6)	N3—C3—Pt1	176.8 (4)
N1^i^—Cs1—C3^viii^	61.52 (10)	N3—C3—Cs1^vii^	70.7 (3)
N1^ii^—Cs1—C3^viii^	165.39 (9)	Pt1—C3—Cs1^vii^	106.37 (15)
N3^vii^—Cs1—C3^viii^	108.55 (9)	N3—C3—Cs2	75.9 (3)
N3^viii^—Cs1—C3^viii^	17.71 (9)	Pt1—C3—Cs2	104.23 (15)
N4^ii^—Cs1—C3^viii^	116.12 (9)	Cs1^vii^—C3—Cs2	110.00 (11)
N4^i^—Cs1—C3^viii^	89.80 (9)	N4—C4—Pt1	178.2 (4)
N2^viii^—Cs1—C3^viii^	60.29 (9)	N4—C4—Cs2	74.1 (3)
N2^vii^—Cs1—C3^viii^	81.03 (9)	Pt1—C4—Cs2	107.38 (14)
C1^ii^—Cs1—C3^viii^	168.91 (9)	N4—C4—Cs1^i^	71.4 (3)
C1^i^—Cs1—C3^viii^	58.23 (9)	Pt1—C4—Cs1^i^	107.30 (14)
N1^ii^—Cs2—N4^i^	92.57 (11)	Cs2—C4—Cs1^i^	144.19 (12)
N1^ii^—Cs2—N3^iii^	70.67 (10)	C1—N1—Cs2^xi^	151.8 (4)
N4^i^—Cs2—N3^iii^	122.44 (10)	C1—N1—Cs1^i^	92.0 (3)
N1^ii^—Cs2—N2^iv^	118.92 (11)	Cs2^xi^—N1—Cs1^i^	93.27 (11)
N4^i^—Cs2—N2^iv^	68.68 (9)	C1—N1—Cs2^vi^	92.8 (3)
N3^iii^—Cs2—N2^iv^	72.87 (11)	Cs2^xi^—N1—Cs2^vi^	107.60 (12)
N1^ii^—Cs2—O1^v^	62.99 (9)	Cs1^i^—N1—Cs2^vi^	121.89 (13)
N4^i^—Cs2—O1^v^	63.17 (8)	C2—N2—Na1^ix^	119.8 (3)
N3^iii^—Cs2—O1^v^	133.63 (9)	C2—N2—Cs2^xii^	140.8 (3)
N2^iv^—Cs2—O1^v^	131.82 (9)	Na1^ix^—N2—Cs2^xii^	95.96 (12)
N1^ii^—Cs2—N4	172.46 (10)	C2—N2—Cs1^vii^	86.4 (3)
N4^i^—Cs2—N4	94.96 (9)	Na1^ix^—N2—Cs1^vii^	95.09 (12)
N3^iii^—Cs2—N4	104.84 (9)	Cs2^xii^—N2—Cs1^vii^	107.33 (11)
N2^iv^—Cs2—N4	64.01 (10)	C2—N2—Cs2^vi^	89.0 (3)
O1^v^—Cs2—N4	121.04 (8)	Na1^ix^—N2—Cs2^vi^	81.26 (11)
N1^ii^—Cs2—N1^ix^	72.40 (12)	Cs2^xii^—N2—Cs2^vi^	80.48 (9)
N4^i^—Cs2—N1^ix^	115.65 (10)	Cs1^vii^—N2—Cs2^vi^	171.76 (12)
N3^iii^—Cs2—N1^ix^	110.69 (10)	C3—N3—Cs2^iii^	133.2 (3)
N2^iv^—Cs2—N1^ix^	168.32 (10)	C3—N3—Cs1^vii^	91.6 (3)
O1^v^—Cs2—N1^ix^	54.15 (9)	Cs2^iii^—N3—Cs1^vii^	112.71 (12)
N4—Cs2—N1^ix^	104.40 (9)	C3—N3—Cs2	86.4 (3)
N1^ii^—Cs2—N3	93.85 (10)	Cs2^iii^—N3—Cs2	113.08 (12)
N4^i^—Cs2—N3	170.13 (9)	Cs1^vii^—N3—Cs2	117.54 (12)
N3^iii^—Cs2—N3	66.92 (12)	C4—N4—Na1	123.3 (3)
N2^iv^—Cs2—N3	114.26 (10)	C4—N4—Cs2^i^	144.7 (3)
O1^v^—Cs2—N3	113.53 (9)	Na1—N4—Cs2^i^	91.37 (11)
N4—Cs2—N3	78.72 (9)	C4—N4—Cs2	87.6 (3)
N1^ix^—Cs2—N3	59.58 (10)	Na1—N4—Cs2	91.55 (11)
N1^ii^—Cs2—C4	155.22 (10)	Cs2^i^—N4—Cs2	85.04 (9)
N4^i^—Cs2—C4	109.89 (10)	C4—N4—Cs1^i^	91.0 (3)
N3^iii^—Cs2—C4	103.78 (10)	Na1—N4—Cs1^i^	97.36 (11)
N2^iv^—Cs2—C4	80.30 (10)	Cs2^i^—N4—Cs1^i^	90.55 (9)
O1^v^—Cs2—C4	117.45 (8)	Cs2—N4—Cs1^i^	170.16 (12)
N4—Cs2—C4	18.33 (9)	Na1—O1—Cs2^xiii^	90.18 (10)
N1^ix^—Cs2—C4	88.03 (9)	Na1—O1—H1A	121 (4)
N3—Cs2—C4	62.56 (9)	Cs2^xiii^—O1—H1A	81 (4)
N1^ii^—Cs2—N2^ix^	122.71 (10)	Na1—O1—H1B	122 (4)
N4^i^—Cs2—N2^ix^	62.33 (9)	Cs2^xiii^—O1—H1B	113 (4)
N3^iii^—Cs2—N2^ix^	166.50 (10)	H1A—O1—H1B	115 (4)
N2^iv^—Cs2—N2^ix^	99.52 (9)	Cs1—O2—Cs2^xiv^	88.88 (9)
O1^v^—Cs2—N2^ix^	59.73 (8)	Cs1—O2—Cs2	88.88 (9)
N4—Cs2—N2^ix^	61.66 (9)	Cs2^xiv^—O2—Cs2	177.76 (18)
N1^ix^—Cs2—N2^ix^	74.63 (9)	Cs1—O2—H2	121 (3)
N3—Cs2—N2^ix^	107.83 (9)	Cs2—O2—H2	147 (4)

Symmetry codes: (i) −*x*+1/2, −*y*+3/2, −*z*; (ii) *x*−1/2, −*y*+3/2, *z*−1/2; (iii) −*x*, −*y*+1, −*z*; (iv) *x*, −*y*+1, *z*−1/2; (v) *x*, *y*+1, *z*; (vi) −*x*+1/2, *y*−1/2, −*z*+1/2; (vii) −*x*, −*y*+2, −*z*; (viii) *x*, −*y*+2, *z*−1/2; (ix) −*x*+1/2, *y*+1/2, −*z*+1/2; (x) −*x*+1/2, −*y*+1/2, −*z*; (xi) *x*+1/2, −*y*+3/2, *z*+1/2; (xii) *x*, −*y*+1, *z*+1/2; (xiii) *x*, *y*−1, *z*; (xiv) −*x*, *y*, −*z*−1/2.

### Hydrogen-bond geometry (Å, °)

**Table:**

*D*—H···*A*	*D*—H	H···*A*	*D*···*A*	*D*—H···*A*
O1—H1A···N1^vi^	0.85 (2)	2.37 (3)	3.154 (6)	155 (5)
O1—H1B···N3^iii^	0.83 (2)	2.17 (2)	2.982 (5)	166 (5)

Symmetry codes: (vi) −*x*+1/2, *y*−1/2, −*z*+1/2; (iii) −*x*, −*y*+1, −*z*.
